# Olfaction and neurocognition after COVID-19: a scoping review

**DOI:** 10.3389/fnins.2023.1198267

**Published:** 2023-06-29

**Authors:** Brandon J. Vilarello, Patricia T. Jacobson, Jeremy P. Tervo, Nicholas A. Waring, David A. Gudis, Terry E. Goldberg, D. P. Devanand, Jonathan B. Overdevest

**Affiliations:** ^1^Columbia University Vagelos College of Physicians and Surgeons, New York, NY, United States; ^2^Department of Otolaryngology-Head and Neck Surgery, New York-Presbyterian/Columbia University Irving Medical Center, New York, NY, United States; ^3^Department of Psychiatry, New York-Presbyterian/Columbia University Irving Medical Center, New York, NY, United States

**Keywords:** COVID, neurocognition, olfaction, review, PASC

## Abstract

**Introduction:**

COVID-19 induces both acute and chronic neurological changes. Existing evidence suggests that chemosensory changes, particularly olfactory loss, may reflect central neurological dysfunction in neurodegenerative diseases and mark progression from mild cognitive impairment to Alzheimer’s. This scoping review summarizes the available literature to evaluate the relationship between neurocognition and olfaction in young to middle-aged adults with minimal comorbidities following COVID-19 infection.

**Methods:**

A literature search of PubMed, Ovid Embase, Web of Science, and Cochrane Library was conducted. Studies underwent title/abstract and full text screening by two reviewers, with a third reviewer resolving any conflicts. Remaining studies underwent data extraction.

**Results:**

Seventeen studies were eligible for data extraction after the review process, where 12 studies found significantly poorer cognition in those suffering from olfactory dysfunction, four studies showed no association between cognition and olfaction, and one study reported lower anosmia prevalence among patients with cognitive impairment.

**Conclusion:**

The majority of studies in this review find that olfactory dysfunction is associated with poorer cognition. More rigorous studies are needed to further elucidate the relationship between olfaction and cognition after COVID-19.

## 1. Introduction

The COVID-19 pandemic propelled olfactory dysfunction to the forefront of otolaryngology research (Hopkins, 2022). Early investigations have provided preliminary insight into the mechanisms by which COVID-19 acutely affects the olfactory system and whether olfaction provides a window into greater neurological dysfunction caused by the virus (Butowt and Bartheld, 2021; Zazhytska et al., 2022). In addition to neurological disturbances of chemosensation, there are numerous reports of other neurological deficits as part of long COVID, also referred to as post-acute sequelae of COVID-19 (PASC). In particular, neurocognitive deficits, frequently referred to as “brain fog,” can persist for more than a year in subsets of patients (Zhou et al., 2020; Hugon et al., 2022; Asadi-Pooya et al., 2023). While many patients report experiencing post-COVID brain fog or memory problems, it is important to note that there are indeed quantifiable structural changes to several areas in the brain (e.g., crus II, cognitive cerebellar lobule) which are associated with greater degrees of cognitive decline in SARS-CoV-2-positive individuals (Douaud et al., 2022). Given that there are discrete structural changes observed in the brain after COVID-19, it is possible that such changes are responsible for specific, measurable cognitive deficits encapsulated within the patient experience of PASC.

The study of olfaction as a biomarker of neurological dysfunction is not new: a body of literature exists that examines the relationship between olfaction and cognitive decline in elderly populations, though studies have shown mixed results. A systematic review found the presence of an association between onset of Alzheimer’s Disease (AD) and olfactory function but highlighted significant variability of study rigor and olfactory testing methodology (Sun et al., 2012). Additionally, the study demonstrated a paucity of prospective, longitudinal study data, calling for further investigation into olfactory testing as a screening tool for AD or mild cognitive impairment (MCI). A recent study showed no statistically significant differences in Sniffin’ Sticks identification scores between individuals with subjective cognitive decline, MCI, and AD (Pusswald et al., 2023).

However, the effects of post-infectious smell loss on neurocognition is not well characterized. The association between olfaction and varying degrees of cognitive impairment are well-documented in the literature among an elderly population; however, as COVID-19-associated olfactory changes are often observed in healthy adults without neurodegenerative changes, synthesizing the body of literature that examines olfaction in healthy young adults is required. Understanding the relationship between olfaction and neurocognition in this population will provide a basis for better understanding the underlying neural processes at work in COVID-19 patients with olfactory changes. Given the associations between olfaction, neurocognition, and COVID-19, we sought to elucidate whether available literature supports olfaction as a biomarker for broader neurological disturbances in PASC among non-elderly, otherwise healthy adults following COVID-19.

## 2. Materials and methods

A systematic literature search of PubMed, Ovid Embase, Web of Science, and Cochrane Library was performed using Preferred Reporting Items for Systematic Reviews and Meta-Analyses extension for Scoping Reviews guidelines (PRISMA-ScR) to capture all studies investigating cognitive outcomes associated with COVID-19-related olfactory dysfunction (Tricco et al., 2018). The search queries used to obtain relevant articles are included in Appendix A.

Articles met inclusion criteria if they were written in English, included adults 18–60 years of age, and examined the associations between olfaction and cognition among a population affected by COVID-19. Articles were excluded if they solely examined a pediatric population ages <18 or elderly population > 60 years, studied individuals with pre-existing neurodegenerative diseases, or were review articles, commentaries, letters to the editor, or conference abstracts. To identify relevant articles, titles and abstracts of each article were screened by two reviewers (BV, PJ, JT, or NW). Articles meeting inclusion criteria after title and abstract review were then screened with full text review by two reviewers. Any disagreements on initial title and abstract or full text review were resolved by a third reviewer that did not perform the initial review. A PRISMA-style flow diagram was generated using Covidence systematic review management software. All articles that passed the full text review then underwent data extraction (Table 1). The primary outcome of interest was the association between olfactory dysfunction related to COVID-19 and cognitive measures. Other data that were extracted from the articles included study author, year published, method of olfaction assessment, method of cognition assessment, and demographic characteristics of patients.

**Table 1:** Data extracted from included studies.

Authors, year	*n*, gender	Mean age ± SD, range	Olfactory assessment methods	Cognition assessment methods	Olfactory results	Cognition results timepoint	Relationship between olfaction and cognition
Alemanno et al. (2021)	87, 25 female	67.23 ± 12.89	Survey	MMSE, MoCA	18/87 had anosmia	Acute respiratory intervention:*intubation:* 74.2% had MoCA deficit, higher than Venturi mask (*p* = 0.005); 12.9% had MMSE deficit, higher than Venturi mask (*p* = 0.024) *BIPAP:* 94.4% had MoCA deficit, 55.6% had MMSE deficit *Venturi mask:* 77.8% had MoCA deficit, 48.3% had MMSE deficit *no O_2_:* 77.8% had MoCA deficit, 44.4% had MMSE deficit	No significant differences in cognitive functions between anosmics and non-anosmics
Almeria et al. (2020)*	35, 19 female	47.6 ± 8.9, 24–60	Retrospective chart review	Digit span (backwards)	20/35 had anosmia	45.7 ± 7.5, 30.0–57.5	Anosmics had lower scores (t = 2.259, *p* = 0.031)
Azcue et al. (2022)	73, 51 female	44.36 ± 9.47, 18–85	BSIT	MoCA, SPCT, SDMT, HVLT-R, BVMT-R, TMT, Benton JLO	53 normal (9.96 ± 0.99), 3 relatively abnormal (7.67 ± 0.58), 15 abnormal (6.47 ± 0.99)	MoCA 25.09 ± 3.06; SPCT-3 16.75 ± 5.26; SDMT 47.23 ± 10.93; HVLT-R trial 1 5.31 ± 1.55, total 22.57 ± 5.92, trial 4 7.90 ± 2.83, DI 9.53 ± 2.47; BVMT-R trial 1 5.63 ± 3.54, trial 1–3 22.38 ± 7.37, trial 4 8.31 ± 2.70, DI 5.64 ± 1.04; TMT-A 38.41 ± 14.50; Benton JLO 24.52 ± 4.98	BSIT showed significant positive correlation with MoCA, SPCT-3, SDMT, HVLT-R trial 1–3, BVMT-R discrimination index, and Benton JLO and significant negative correlation with TMT-A. Participants with abnormal BSIT had significantly worse general cognition, attention, verbal memory, visual memory, visuospatial perception, and abstraction capacity.
Cacciatore et al. (2022)	83, 20 females	66.9, 95% CI: 64.2–69.7	Survey	MoCA	15/83 had hyposmia/hypogeusia	Mean 24.1, range 23.4–24.8	No significant correlation between cognition and hyposmia/hypogeusia
Caspersen et al. (2022)	774, 449 female	25–65+	Survey	Survey	COVID-19 dx 11–12 months ago: 28 had altered smell or taste COVID-19 dx 1–6 months ago: 128 had altered smell or taste	COVID-19 dx 11–12 months ago: 30 had poor memory, 20 had brain fog COVID-19 dx 1–6 months ago: 81 had poor memory, 84 had brain fog	No significant correlation between altered smell or taste and poor memory or brain fog
Cecchetti et al. (2022)	49, 13 females	60.8 ± 12.6	Survey	Phonemic fluency, SDMT, RAVLT immediate recall	22/49 had dysgeusia/hyposmia during acute COVID-19	baseline: phonemic fluency 27.9 ± 10.2, SDMT 35.1 ± 1.9, RAVLT 29.3 ± 9.4 follow up: phonemic fluency 31.9 ± 11.4, SDMT 41.8 ± 1.3, RAVLT 35.8 ± 11.2	Those with dysgeusia/hyposmia had less RAVLT (immediate recall memory) improvement; no significant difference in improvement on phonemic fluency or SDMT
Chen et al. (2022)	200, 129 females	44.6 ± 15.46, 19–82	Survey, UPSIT	MoCA, NIH Toolbox	Survey: 109/200 had smell changes UPSIT: 53/164 had normosmia, 62/164 had mild hyposmia, 32/164 had moderate hyposmia, 13/164 had severe hyposmia, 4/164 had anosmia	102/191 had normal MoCA, 89/191 had cognitive impairment; for NIH-TB language, 138/196 had >25% and 58 had ≤25%; for NIH-TB working memory, 134/196 had >25% and 62/196 had ≤25%	Weak correlation between UPSIT and MoCA (r = 0.30, *p* = 0.0002); weak correlation between UPSIT and NIH-TB language (r = 0.36, *p* < 0.0001)
Damiano et al. (2023)	701, 334 females	55.3 ± 14.6, 95% CI: 54.3–56.3	Survey	MCS, MMSE, TMT, DSST, Neuropsychological Battery CERAD	9% had parosmia, 18% had moderate and severe olfactory deficits	MCS 5.2 ± 4.16, MMSE orientation 8.27 ± 3.25, TMT-A 65.5 ± 48.0, verbal fluency 15.57 ± 5.43, DSST 32.3 ± 19.3, Boston naming 13.15 ± 2.27, word list 15.35 ± 4.7, construction praxis 8.26 ± 2.55, word list recall 4.86 ± 2.25, word list recognition 7.88 ± 2.77	Parosmia significantly associated with MCS (*p* = 0.001) and Boston naming (*p* = 0.017); moderate & severe olfactory deficit associated with TMT-A (*p* = 0.008), digit-symbol (*p* = 0.009), word list memory task (*p* = 0.041)
Delgado-Alonso et al. (2022)	50, 37 females	51.06 ± 11.65	BSIT	Digit span (backwards), ROCF, Stroop A, inhibition test, determination test, divided attention, selective attention, FGT	9.00 ± 2.33	Frequency of impairment 2x more than expected for digit span, ROCF (memory at 30 min); frequency of impairment at least 3x more than expected for Stroop A; inhibition test 7.74 ± 3.91, determination test 198.31 ± 48.63, divided attention 561.37 ± 216.40, selective attention 429.66 ± 124.86, FGT Delayed Free Recognition I 5.70 ± 2.99	BSIT showed moderate correlations with digit span (backwards) (R = 0.505), ROCF (memory at 30 min) (R = 0.383), Stroop A (R = 0.387), inhibition test (R = -0.374), determination test (R = 0.36), divided attention (R = 0.335), selective attention (R = -0.318), and FGT (Delayed Free Recognition I) (R = 0.347)
Desai et al. (2022)	49, 36 female	18–76	Survey, UPSIT	CNS Vital Signs validated cognitive remote testing website, neurocognitive index, composite memory, verbal memory, visual memory, psychomotor speed, reaction time, complex attention, cognitive flexibility, processing speed, executive function, simple attention, motor speed	Survey: *active COVID-19:* 13% had anosmia and 50% had hyposmia *recovered:* 4% had anosmia and 67% had hyposmia UPSIT: *active COVID-19:* 37.5% had anosmia, 18.75% had hyposmia, 43.75% had normosmia *recovered:* 33.33% had anosmia, 46.67%, had hyposmia, 20% had normosmia	Active vs. recovered cognitive flexibility 48.9 vs. 34.8, complex attention 46.6 vs. 49.0, composite memory 42.7 vs. 43.8 executive fxn 52.4 vs. 34.7, motor speed 49.1 vs. 43.2, neurocognitive index 47.1 vs. 35.8, processing speed 57.5 vs. 42.0; rxn time 49.0 vs. 31.0, simple attn. 46.4 vs. 49.8, verbal memory 43.1 vs. 45.9, visual memory 45.3 vs. 45.8	No correlation between self-reported smell loss and cognitive function; nonsignificant inverse association between UPSIT score and processing speed in recovered; no correlations with cognitive percentiles and UPSIT total scores
Di Stadio et al. (2022)	152, 102 females	41.2 ± 11, 18–65	Sniffin’ Sticks identification	MMSE, survey	50/152 had anosmia, 25/152 had hyposmia, 10/152 had parosmia/cacosmia, 58/152 had combination of hyposmia and parosmia	MMSE wnl; 23.7% reported mental clouding	Patients with mental clouding had higher risk of suffering from anosmia (OR 19, p = 0.05), hyposmia + parosmia (OR 33, p = 0.01), hyposmia alone (OR 15, *p* = 0.07), and moderate risk of suffering from parosmia (OR 3, *p* = 0.5) compared to patients with no neurological symptoms
Ferrucci et al. (2022)	Time_1_: 76, 20 females; Time_2_: 53, 15 females	56.24 ± 12.08, 18–75	Survey	BRB-NT, SRT, SPART, SDMT, PASAT, WLG	Time_1_: 44.6% had hyposmia, 42.1% had hyposmia and dysgeusia Time_2_: 9.4% had hyposmia	SPART-D = 5.66 ± 2.07, SRT-LTS = 35.64 ± 13.77, SRT-CLTR = 27.75 ± 13.06, SRT-D = 6.92 ± 2.66, SPART = 17.75 + 5.01, SDMT = 38.81 ± 9.88, PASAT-3 = 41.66 ± 11.98, PASAT2 = 30.81 ± 9.36, WLG = 24.75 ± 4.69	SPART-D (delayed visuospatial memory recall) score worse in those who reported hyposmia
Fiorentino et al. (2022)*	84, 55 females	42.8 ± 13.6, 19–59	Sniffin’ Sticks, TODA	PPTT, generic naming test from Grémots battery: Evaluation du langage dans les pathologies neurodégénératives	Sniffin’ Sticks: *age 19–39:* T 4.76 ± 4.04, D 9.51 ± 3.84, I 9.40 ± 3.92, TDI 23.68 ± 9.68 *age 40–59:* T 4.25 ± 3.26, D 9.55 ± 3.86, I 10.15 ± 3.52, TDI 23.95 ± 8.61 TODA *age 19–39:* threshold 1.66 ± 0.97, identification 3.97 ± 1.76 *age 40–59:* threshold 1.41 ± 0.87, identification 4.19 ± 1.63	Age 19–39: PPTT 47.31 ± 2.63; generative naming strict 34 ± 2, broad 34 ± 1, time 63.93 ± 17.51 age 40–59: PPTT 49.20; generative naming strict 34 ± 1, broad 35 ± 1, time 59.46 ± 16.34	For PPTT and TODA T, small significant correlation between semantic memory and odor threshold detection
Jennings et al. (2022)	108, 76 females	46.3 ± 10.3, 25–78	Survey	Survey	21/108 had dysosmia	71 had brain fog, 37 did not have brain fog	25.4% of participants with brain fog reported dysosmia, 8.1% of participants without brain fog reported dysosmia; in cluster analysis, dysosmia was more prevalent in the brain fog group in a two-cluster model
Kopishinskaia et al. (2021)	187, 152 females	35, 21–87	Survey	Survey	All patients had parosmia/phantosmia	40/187 had brain fog	Brain fog was significantly higher in patients with parosmia/phantosmia compared to controls
Llana et al. (2022)*	42, 38 females	31–51	Survey	MTT, PAL, MoCA, DSST	17/42 had anosmia, 25/42 did not have anosmia	MTT ERI-d2: anosmics −9.01 ± 55.78, non-anosmics −49.81 ± 44.19; MoCA: anosmics 25.81 ± 2.42, non-anosmics 26.39 ± 2.64, controls 28.07 ± 1.58; DSS-M: anosmics 64.50 ± 16.08, controls 84.59 ± 10.64; DSS-IL anosmics 10.19 ± 4.79, controls 13.41 ± 3.71; DSS-R anosmics 6.81 ± 1.47, controls 7.83 ± 1.04	For MTT, ERI-d2 index was Higher in anosmics than non-anosmics with COVID-19; anosmics had lower scores on MoCA and DSST than controls without COVID-19
Tavares-Júnior et al. (2022)	141, 89 females	48 ± 14, 16–90	Survey	ACE-R, MMSE, CDR	Normal cognition: 19 had anosmia, 29 did not Cognitive impairment:1 had anosmia, 24 did not Subjective cognitive decline: 23 had anosmia, 45 did not	48 had normal cognition, 25 had cognitive impairment, 68 had subjective cognitive decline	Cognitive impairment group had a lower frequency of anosmia than the normal and subjective cognitive decline groups

*These studies contain only participants between ages 18 and 60.

ACE-R, Addenbrooke’s cognitive examination-revised; BRB-NT, brief repeatable battery of neuropsychological tests; BVMT-R, brief visuospatial memory test-revised; CDR, clinical dementia rating; DSS-IL, digit symbol substation incidental learning; DSS-M, digit symbol substation correctly matched; DSS-R, digit symbol substation incidental learning registered; DSST, digit symbol substitution test; FGT, figural memory test; HVLT-R, Hopkins verbal learning test-revised; JLO, judgment line orientation test; MCS, memory complaint scale; MMSE, mini-mental state examination; MoCA, Montreal cognitive assessment; MTT, mirror tracing test; MTT ERI-d2, mirror tracing test consolidation of learning; PAL, paired-associated learning; PASAT, paced serial additions test; PPTT, pyramids and palm trees test; RAVLT, Rey auditory verbal learning test; ROCF, Rey-Osterrieth complex figure; SDMT, symbol digit modality test; SPART, spatial recall test; SPCT, Salthouse perceptual comparison test; SRT, serial recall test; TMT, trail making test; WLG, word list generation.

## 3. Results

### 3.1. Review process

**Figure 1:**
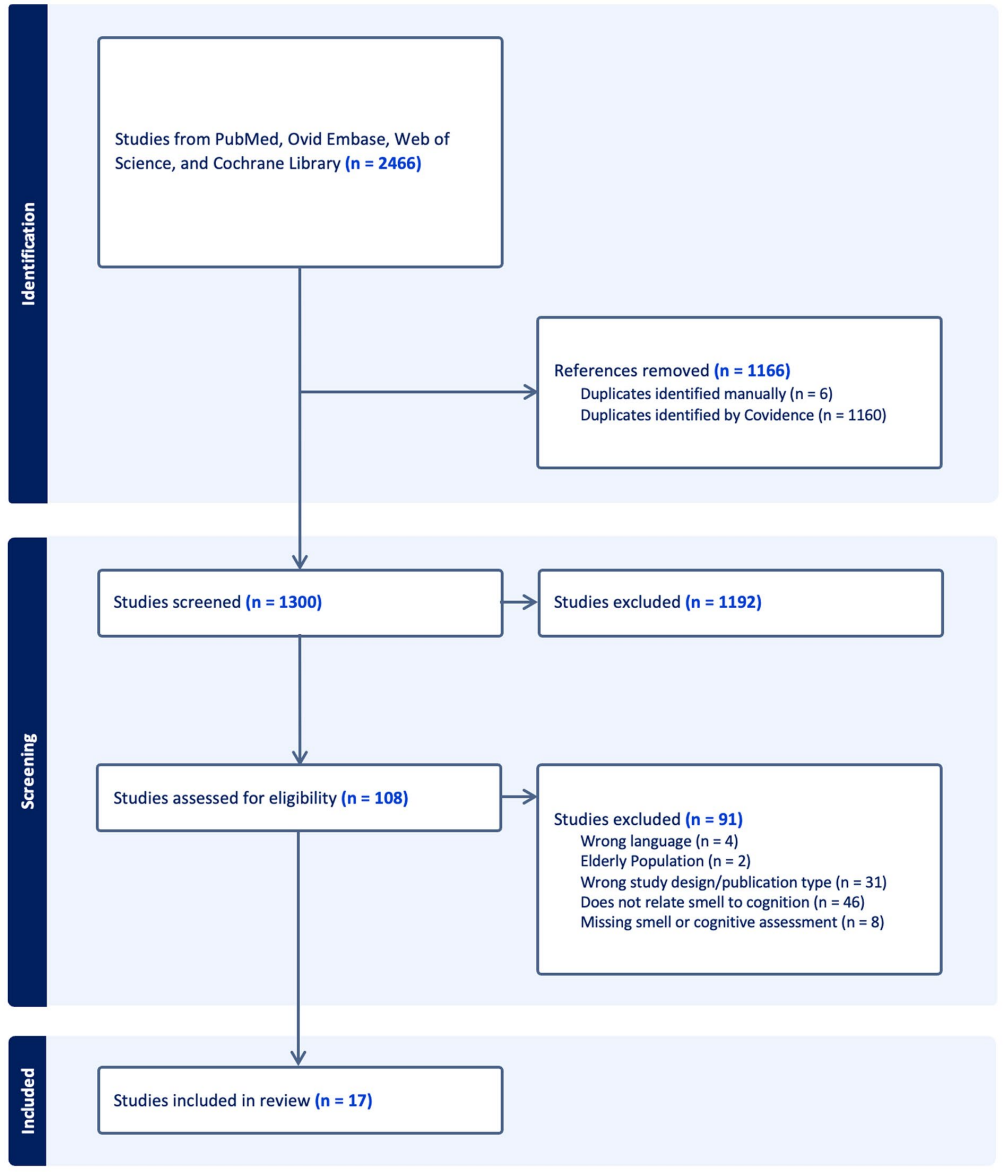
PRISMA-style flow chart documenting the study screening process.

We conducted our systematic review of available literature in January 2023, which yielded 2,466 articles. Removal of 1,166 duplicates resulted in a total of 1,300 articles for title and abstract screening. Of these, 108 articles moved on to full text and bibliographic reference review, where 91 studies were excluded for the following reasons: wrong language, wrong population age, wrong study design or publication type, failure to include assessment of olfaction or cognition, and failure to directly analyze the association between olfaction and cognition. Conclusion of this process resulted in 17 studies eligible for data extraction and inclusion in our review. Figure 1 illustrates the PRISMA-style flow chart documenting the study screening process.

### 3.2. Study participants

All studies that underwent data extraction include young to middle-aged adults. Notably, all but three of these studies included cohort population results for elderly adults; a single study included at least one adolescent in addition to the target population. Twelve studies have more females than males; and, among studies reporting explicit ages, the mean age of the extracted population data range from 35 to 67.23 with standard deviations ranging from 8.9 to 15.46.

### 3.3. Assessment of olfaction

Methods for assessing olfaction included both subjective self-report and psychophysical (semi-objective) assessments of olfaction. Subjective methods were survey (12/17 studies) and chart review (1/17 studies). Seven studies incorporated psychophysical assessment methods, including Brief Smell Identification Test (BSIT) (2/17 studies), University of Pennsylvania Smell Identification Test (UPSIT) (2/17 studies), Sniffin’ Sticks (2/17 studies), and *Test Olfactif informatisé pour le Diagnostic de la maladie d’Alzheimer et de l’Apathie* (TODA) (1/17 studies). Only one study lacked experimental groups composed of both patients with and without smell dysfunction. A single study had an experimental group composed of only individuals with qualitative smell changes (Kopishinskaia et al., 2021). Most studies examined primarily quantitative smell loss, while three studies assessed qualitative smell alteration.

Of the 17 studies included in the review, 11 studies utilized subjective reports only to assign olfactory status. Among the studies using subjective reports as the measure of olfactory function, three studies did not find any significant difference in cognition between normosmics and those with smell loss (Alemanno et al., 2021; Cacciatore et al., 2022; Caspersen et al., 2022). Seven studies found that those with smell loss had worse cognition than those without (Almeria et al., 2020; Kopishinskaia et al., 2021; Cecchetti et al., 2022; Ferrucci et al., 2022; Jennings et al., 2022; Llana et al., 2022; Damiano et al., 2023), and one study found that those with smell loss had better cognition (Tavares-Júnior et al., 2022). This discrepancy was resolved among results from studies that utilized a psychophysical assessment of olfaction. In these six studies, five reported significantly worse cognitive performance in the smell loss group (Azcue et al., 2022; Chen et al., 2022; Delgado-Alonso et al., 2022; Di Stadio et al., 2022; Fiorentino et al., 2022), with the remaining study reporting no significant difference (Desai et al., 2022).

### 3.4. Assessment of cognition

Methods for assessing cognition are widely heterogenous in these studies and consist of both self-report and clinical assessment. Some cognitive tests examine general cognition through screening tools such as the Montreal cognitive assessment (MoCA) or mini-mental state examination (MMSE), while other tests focus on specific cognitive domains. Assessment methods utilized in 3 or more studies include MoCA, MMSE, symbol digit modality test (SDMT), and generalized survey instruments.

There were no apparent patterns for certain cognitive tests to associate with significant findings, other than those including a factor to evaluate memory. Memory, including working, verbal, visual, and semantic memory, was specifically tested in nine of the studies. Of these, seven studies found that those with olfactory dysfunction had significantly worse memory than those without olfactory dysfunction (Azcue et al., 2022; Cecchetti et al., 2022; Chen et al., 2022; Delgado-Alonso et al., 2022; Ferrucci et al., 2022; Fiorentino et al., 2022; Damiano et al., 2023), while two studies found no significant difference (Caspersen et al., 2022; Desai et al., 2022).

As a caveat, 4/17 studies used a survey to assess patient cognition; two of these studies showed a significant relationship between olfaction and cognition, where olfaction was also only assessed with a self-report survey (Kopishinskaia et al., 2021; Jennings et al., 2022). The study by Caspersen et al. (2022) similarly utilized surveys to assess both cognition and olfaction, though this study did not find any significant associations between them. A single study (Di Stadio et al., 2022) used both survey and MMSE to assess cognition of participants and showed a significant relationship between survey outcomes (e.g., mental clouding) and olfactory dysfunction. However, the MMSE data for Di Stadio et al. (2022) showed that participants scored an average that was within normal limits.

### 3.5. Relationship between olfaction and cognition

Thirteen of the included studies demonstrated a significant association between olfaction and cognition, with all but one of these suggesting lower cognitive performance among those with olfactory dysfunction (Almeria et al., 2020; Kopishinskaia et al., 2021; Azcue et al., 2022; Cecchetti et al., 2022; Chen et al., 2022; Delgado-Alonso et al., 2022; Di Stadio et al., 2022; Ferrucci et al., 2022; Fiorentino et al., 2022; Jennings et al., 2022; Llana et al., 2022; Damiano et al., 2023). However, one study reported a lower frequency of anosmia in those with cognitive impairment compared to controls (Tavares-Júnior et al., 2022), and four studies found no statistically significant association between olfaction and cognition (Alemanno et al., 2021; Cacciatore et al., 2022; Caspersen et al., 2022; Desai et al., 2022).

### 3.6. Impact of severity of smell loss on cognition

Results from these studies are in-line with a dose-dependent relationship between the severity of olfactory dysfunction and neurocognitive deficits. Of the six studies that used psychophysical olfactory testing, which can detect varying levels of olfactory deficit severity, five found positive correlations between scores of olfaction and neurocognition. Azcue et al. (2022) and Delgado-Alonso et al. (2022) found statistically significant positive correlations between the BSIT and multiple tests of cognition. Chen et al. (2022) found a positive correlation between the UPSIT and the MoCA and NIH-TB tests. Fiorentino et al. (2022) used the TODA test of olfaction and PPTT test of cognition and found a positive correlation between odor detection threshold and semantic memory. The Di Stadio et al. (2022) study used the Sniffin’ Sticks test to stratify olfactory deficit severity into categories of normosmia, hyposmia, and anosmia. Although all of the patients in that study scored in the normal range in the MMSE, patients who reported subjective mental clouding had a greater odds of anosmia (OR 19, *p* = 0.05) and hyposmia alone (OR 15, *p* = 0.07), though neither of these achieved statistical significance. In contrast to the other five studies using semi-objective olfactory assessments, Desai et al. (2022) found no apparent correlation between olfaction (assessed by UPSIT) and measures of cognition across patients actively infected with COVID-19 and those recovered from COVID-19. Interestingly, they found an inverse correlation between UPSIT scores and processing speed specifically in the COVID-19 recovered patients, though this relationship was not statistically significant (*p* = 0.122). However, unique to this study, the UPSIT test was self-administered rather than proctored.

## 4. Discussion

### 4.1. Summary of findings

A statistically significant association between olfactory deficits and poorer cognition was reported in 12 of 17 studies. Four studies found no association between olfaction and cognition. One study noted lower anosmia frequency in those with cognitive impairment. The methods used to assess olfaction and cognition were heterogenous and included both subjective self-reported measures and psychophysical clinical assessments.

### 4.2. Methods of olfactory assessment

Although patient report is the least time-consuming method for assessing olfactory status, it has been shown to consistently offer a less accurate measurement of olfaction (Philpott et al., 2006). Variability in reported associations between olfaction and cognition could thus reflect a decreased reliability of self-reported olfactory status. It is important to note that there are several components contributing to an individual’s olfaction; namely, the presence of olfactory threshold, discrimination, and identification, where these complementary domains help to parse out specific pathways that contribute to the sense of smell (Hummel et al., 1997). Importantly, these domains localize to specific components of olfactory detection and processing. Olfactory threshold primarily represents the peripheral olfactory system whereas discrimination and identification may represent higher cognitive processing and, unlike olfactory threshold, are frequently unchanged in states of sinonasal disease (Hedner et al., 2010; Oleszkiewicz et al., 2019). All of the studies included in this scoping review used either survey, retrospective chart review, or olfactory identification testing to elucidate olfactory status among subjects, suggesting a need to evaluate individuals with persistent olfactory dysfunction following COVID using a more comprehensive examination of threshold, discrimination, and identification alongside cognitive testing to fully understand the impact of PASC on these domains.

### 4.3. Methods of cognitive assessment

Among studies that focused on assessment of memory as a cognitive domain, seven of nine identified worse outcomes in memory test results among those with olfactory dysfunction compared to those without. Interestingly, prior studies in patients with Alzheimer disease have demonstrated that impaired olfactory identification may predict an individual’s memory decline (Zou et al., 2016; Yu et al., 2018). The relationship between olfactory dysfunction and diminished memory may be due to the role that one’s sense of smell has on memory formation (Herz, 2016; Bruijn and Bender, 2018). In contrast, the use of self-reported cognitive assessments via survey produced more heterogeneous results when examining the relationships between olfaction and cognition. Even within the results of one study itself (Di Stadio et al., 2022), there were mixed results with the use of self-report versus objective measurements of cognition. This discrepancy in cognition between objective normalcy and subjective dysfunction highlights the difficulty of drawing conclusions from patient-reported data.

### 4.4. Parosmia and cognition

Qualitative smell loss (parosmia) appears to have a unique impact on patient cognitive domains in comparison to quantitative smell loss (hyposmia/anosmia). The study by Di Stadio et al. (2022) describes that parosmia has a small and statistically insignificant impact on subjective patient reports of mental clouding. At the same time, Damiano et al. (2023) found parosmia to have a significant association with post-COVID-19 patient scores on the Memory Complaint Scale (MCS), a subjective measure of one’s perceived cognitive abilities. They also show that parosmia is significantly associated with objective neuropsychiatric morbidity via lower scores on the Boston Naming Test, an objective assessment of visual confrontation naming, language, communication, memory, and problem-solving processes. Interestingly, Di Stadio et al. (2022) found that patients with a combination of hyposmia and parosmia had the highest odds of reporting mental clouding. The disparate findings between these studies may indicate that the patients who report parosmia and are later found to be hyposmic on semi-objective olfactory assessment are at the greatest risk for measurable neuropsychiatric impairment. The disparate effects that parosmia and hyposmia/anosmia have on neurocognition could be explained by varying degrees of neuroinvasion, downregulation of olfactory receptors, or possibly due to an overlap of these phenomena leading those with semi-objectively assessed smell loss to have more robust cognitive changes than those with subjective smell loss alone (Yachou et al., 2020; Zazhytska et al., 2022).

### 4.5. Global relationship between olfaction and cognition

There are many studies which have examined the general relationship between olfaction and cognition with the majority showing that olfactory performance tends to have significant associations with measurements of frontal lobe executive function (Westervelt et al., 2005; Challakere Ramaswamy and Schofield, 2022; Mattos et al., 2022). There are a variety of medical conditions in which there is evidence for a positive association between olfactory performance and cognitive functioning. Some of the most robust findings for this correlation have been shown in neurodegenerative, multiple sclerosis, psychiatric, and traumatic brain injury (TBI) populations (Devanand et al., 2010; Challakere Ramaswamy and Schofield, 2022). Several of these conditions have a strong basis for concurrent disease mechanisms causing dysfunction in both cognition and olfaction. For example, TBI commonly affects the frontal lobe during rapid acceleration/deceleration head injury, which can lead to executive function deficits (Rabinowitz and Levin, 2014). Simultaneously, TBI can lead to olfactory dysfunction through sinonasal tract disruption, shearing of the olfactory nerve, or contusion of olfactory bulb and cortex (Howell et al., 2018). In the case of neurodegenerative disease, patients with Lewy bodies (e.g., Alzheimer’s disease with predominantly limbic Lewy bodies) have diffuse proteinopathy that most commonly affects the olfactory bulb along with other brain regions leading to olfactory and cognitive impairment (Beach et al., 2009).

Unlike states of trauma or neurodegeneration, the mechanism linking olfactory dysfunction to cognitive deficits in a younger, healthy patient population is less understood. Recently, there have been studies suggesting that humans have an intrinsic association between olfactory identification and spatial memory even outside of disease states (Dahmani et al., 2018). Given this association, a natural question is whether declines in olfactory function could independently contribute to cognitive deficits. A significant body of research early in the COVID-19 pandemic focused on the possibility of SARS-CoV-2’s ability to directly invade the central nervous system through the olfactory mucosa and olfactory nerve (Kumari et al., 2021; Meinhardt et al., 2021). However, there is now substantial evidence indicating that these studies may have simply identified residual SARS-CoV-2 spike proteins within the brain without identifying the virus itself (Butowt et al., 2021). Additionally, there is now a strong model showing that early-stage SARS-CoV-2-induced anosmia stems from altering the function of olfactory sensory neurons rather than through direct infection (Zazhytska et al., 2022). Therefore, the growing evidence of COVID-related olfactory deficits without signs of direct neuroinvasion suggests that the mechanism linking post-COVID olfactory dysfunction with cognitive deficits could be related to the intrinsic association between olfaction and cognition in humans.

### 4.6. Limitations

This study is not without limitations. Out of the 17 studies, only three studies provided a population exclusive to individuals between 18 and 60 years of age. The remaining 14 studies included more heterogeneity in the age of their study populations and included participants who were older than 60 years. As individuals age, not only are they at higher risk for neurodegenerative diseases such as dementia, but also their cognitive functioning in certain areas such as processing speed and working memory may also decline (Harada et al., 2013; Murman, 2015). Additionally, there is a pronounced decrease in olfactory performance among people ages 60–71 years (Oleszkiewicz et al., 2019).

The studies included in this review each contained highly variable numbers of participants, where the disparate quantities of study participants add complexity when comparing the strength of findings between papers. For example, there are high-powered studies including several with 100+ participants that show disparate conclusions regarding correlations between olfaction and cognition. The Caspersen et al. (2022) study included 774 participants and found that there was no significant correlation between olfaction and cognition after COVID. At the same time, the Damiano et al. (2023) study included 701 participants and found there to be highly significant associations between olfaction dysfunction and worse cognitive performance in several cognitive tests. The disparities between these studies could be attributed to their varying methods for assessing both olfaction and cognition. Specifically, the use of subjective, survey-based assessments for olfaction (11/17 studies) and cognition (4/17 studies) limits the strength of objective conclusions on the relationship between these domains. Another limitation is the varying frequency of olfactory impairment among populations included, as some studies showed that nearly half of the participants exhibited olfactory dysfunction (Desai et al., 2022; Ferrucci et al., 2022), while others show that only ~20% of participants screened positive for olfactory dysfunction (Alemanno et al., 2021; Azcue et al., 2022; Cacciatore et al., 2022; Jennings et al., 2022). Additional studies utilizing psychophysical assessments of both olfaction and cognition along with larger numbers of participants with post-COVID olfactory dysfunction will help to better understand the effect COVID-19-related olfactory dysfunction has on cognitive performance.

## 5. Conclusion

The majority of studies in this review find that olfactory dysfunction is associated with poorer cognition, consistent with prior research in the area of neurodegenerative diseases, but a unique finding for post-infectious olfactory dysfunction. Despite these findings, studies that include individuals of highly variable ages fail to fully isolate the effects of aging on olfaction and cognition. Additional longitudinal, prospective studies are needed to understand how olfaction provides a window into the central nervous system in individuals affected by acute and chronic sequelae of COVID-19.

## Author contributions

BV and PJ created and revised the search terms. BV, PJ, JT, and NW all served as independent reviewers for the title, abstract and full-text screening processes and wrote the article. DG, TG, DD, and JO conceptualized the topic and themes for this manuscript, provided direct supervision of the review process, and provided edits and revisions for the manuscript. All authors contributed to the article and approved the submitted version.

## Funding

This work was supported by grant K23DC019678 (author JO) from the National Institute on Deafness and Other Communication Disorders (https://www.nih.gov/about-nih/what-we-do/nih-almanac/national-institute-deafness-other-communication-disorders-nidcd) and the National Institutes of Health. The content is solely the responsibility of the authors and does not necessarily represent the official views of the NIH. The funders did not play any role in study design, data collection/analysis, decision to publish, or manuscript preparation.

## Conflict of interest

The authors declare that the research was conducted in the absence of any commercial or financial relationships that could be construed as a potential conflict of interest.

## Publisher’s note

All claims expressed in this article are solely those of the authors and do not necessarily represent those of their affiliated organizations, or those of the publisher, the editors and the reviewers. Any product that may be evaluated in this article, or claim that may be made by its manufacturer, is not guaranteed or endorsed by the publisher.

## Appendix A: Search Terms

PubMed (864 results)

((“Smell”[MeSH] OR “olfaction disorders”[MeSH] OR “Anosmia”[MeSH] OR smell [tiab] OR olfact*[tiab] OR phantosmia*[tiab] OR parosmia*[tiab] OR hyposmi*[tiab] OR anosmi*[tiab] OR cacosmi*[tiab] OR dysosmi*[tiab]) AND (“Cognitive dysfunction”[MeSH] OR “Cognition”[MeSH] OR cogniti*[tiab] OR “brain function*”[tiab] OR neurolog*[tiab] OR “mental status*”[tiab] OR memor*[tiab] OR “executive dysfunction*”[tiab]) AND (“COVID-19” [MeSH] OR “SARS-CoV-2” [MeSH] OR “Post-Acute COVID-19 Syndrome” [MeSH] OR COVID [tiab] OR “SARS-CoV-2”[tiab] OR nCoV [tiab]))

Ovid Embase (1,465 results)

((smelling/ or smelling disorder/ or anosmia/ or (smell or olfact* or phantosmia* or parosmia* or hyposmi* or anosmi* or cacosmi* or dysosmi*).tw.) and (cognitive defect/ or cognition/ or (cogniti* or brain function* or neurolog* or mental status* or memor* or executive dysfunction*).tw.) and (coronavirus disease 2019/or (COVID or “SARS-CoV-2” or nCoV).tw.))

Web of Science (Core Collection – Clarivate) (838 results)

TS = ((smell OR olfact* OR phantosmia* OR parosmia* OR hyposmi* OR anosmi* OR cacosmi* OR dysosmi*) AND (cogniti* OR brain function* OR neurolog* OR mental status* OR memor* OR executive dysfunction*) AND (COVID OR “SARS-CoV-2” OR nCoV))

Cochrane Library (71 results)

((smell OR olfact* OR phantosmia* OR parosmia* OR hyposmi* OR anosmi* OR cacosmi* OR dysosmi*) AND (cogniti* OR brain function* OR neurolog* OR mental status* OR memor* OR executive dysfunction*) AND (COVID OR “SARS-CoV-2” OR nCoV))
